# Dyschloremia is associated with failure to restore renal function in survivors with acute kidney injury: an observation retrospective study

**DOI:** 10.1038/s41598-020-76798-5

**Published:** 2020-11-12

**Authors:** Youn Kyung Kee, Hee Jung Jeon, Jieun Oh, Dong Ho Shin

**Affiliations:** Department of Internal Medicine, College of Medicine, Kangdong Sacred Heart Hospital, Hallym University, 150, Seongan-ro, Gangdong-gu, Seoul, 05355 Korea

**Keywords:** Medical research, Nephrology

## Abstract

Dyschloremia is common in critically ill patients. However, little is known about the effects of dyschloremia on renal function in patients with acute kidney injury (AKI) requiring continuous renal replacement therapy (CRRT). A total of 483 patients who received CRRT for AKI were selected and divided into three groups according to their serum chloride concentrations at the time of CRRT initiation. At 90 days after initiating CRRT, renal outcome, i.e., non-complete renal recovery, or renal failure, was assessed in the three groups. The hypochloremia group (serum chloride concentrations < 96 mEq/L, n = 60), the normochloremia group (serum chloride concentrations, 96–111 mEq/L, n = 345), and the hyperchloremia group (serum chloride concentrations > 111 mEq/L, n = 78) were classified. The simplified acute physiology score III was higher in the hyperchloremia and hypochloremia groups than in the normochloremia group. Multivariate logistic regression analyses showed that hypochloremia (odds ratio, 5.12; 95% confidence interval [CI], 2.56–10.23; *P* < 0.001) and hyperchloremia (odds ratio, 2.53; 95% CI, 1.25–5.13; *P* = 0.01) were significantly associated with non-complete renal recovery. Similar trends were observed for renal failure. This study showed that dyschloremia was independently associated with failure in restoring renal function following AKI.

## Introduction

Severe acute kidney injury (AKI) requiring renal replacement therapy (RRT) is a common serious complication in critically ill patients and is associated with high mortality and morbidity^1–4^. Although critical care and dialysis technology have improved significantly, the mortality in patients with severe AKI requiring RRT is higher than in those with other serious diseases, such as acute respiratory distress syndrome or myocardial infarction^5,6^. In addition, survivors with severe AKI requiring RRT are known to have a high risk of developing long-term complications, such as chronic kidney disease (CKD) or renal failure^4^.

Chloride is a major extracellular anion in the blood which constitutes approximately one-third of extracellular fluid tonicity^7,8^. In addition, it plays several important roles in the body, such as maintaining electrical activity, acid–base balance, fluid and gastrointestinal homeostasis, and renal function^7,9–11^. Despite its physiological and clinical importance, less attention has been paid to chloride than other routinely measured electrolytes in critically ill patients^8^. Dyschloremia commonly observed in critically ill patients can occur because of various etiologic factors associated with the illness or treatment^7,12,13^. Although there have been few outcome-related studies on dyschloremia, several studies showed that dyschloremia is associated with significantly increased mortality and morbidity^11,14–16^. Some studies have shown that hyperchloremia could reduce renal blood flow and glomerular filtration rate (GFR) and consequently, cause salt and water retention to occur^17,18^. These findings suggest that hyperchloremia is associated with AKI based on clear biological plausibility. In fact, accumulation of clinical evidence also suggests that hyperchloremia is associated with AKI in critically ill patients^19,20^. Although there is no clear experimental evidence for hypochloremia-associated AKI, some observational clinical studies have shown that hypochloremia is associated with AKI^21,22^. Moreover, renal recovery following AKI is clinically important because AKI had been found to be an independent risk factor for the development of CKD or the progression from CKD to renal failure^23^. However, there is limited knowledge regarding the association between serum chloride concentrations and renal recovery in patients with AKI. Thus, this study aimed to determine whether dyschloremia was associated with failure to restore renal function in survivors having severe AKI and requiring RRT.

## Results

### Study population

**Figure 1:**
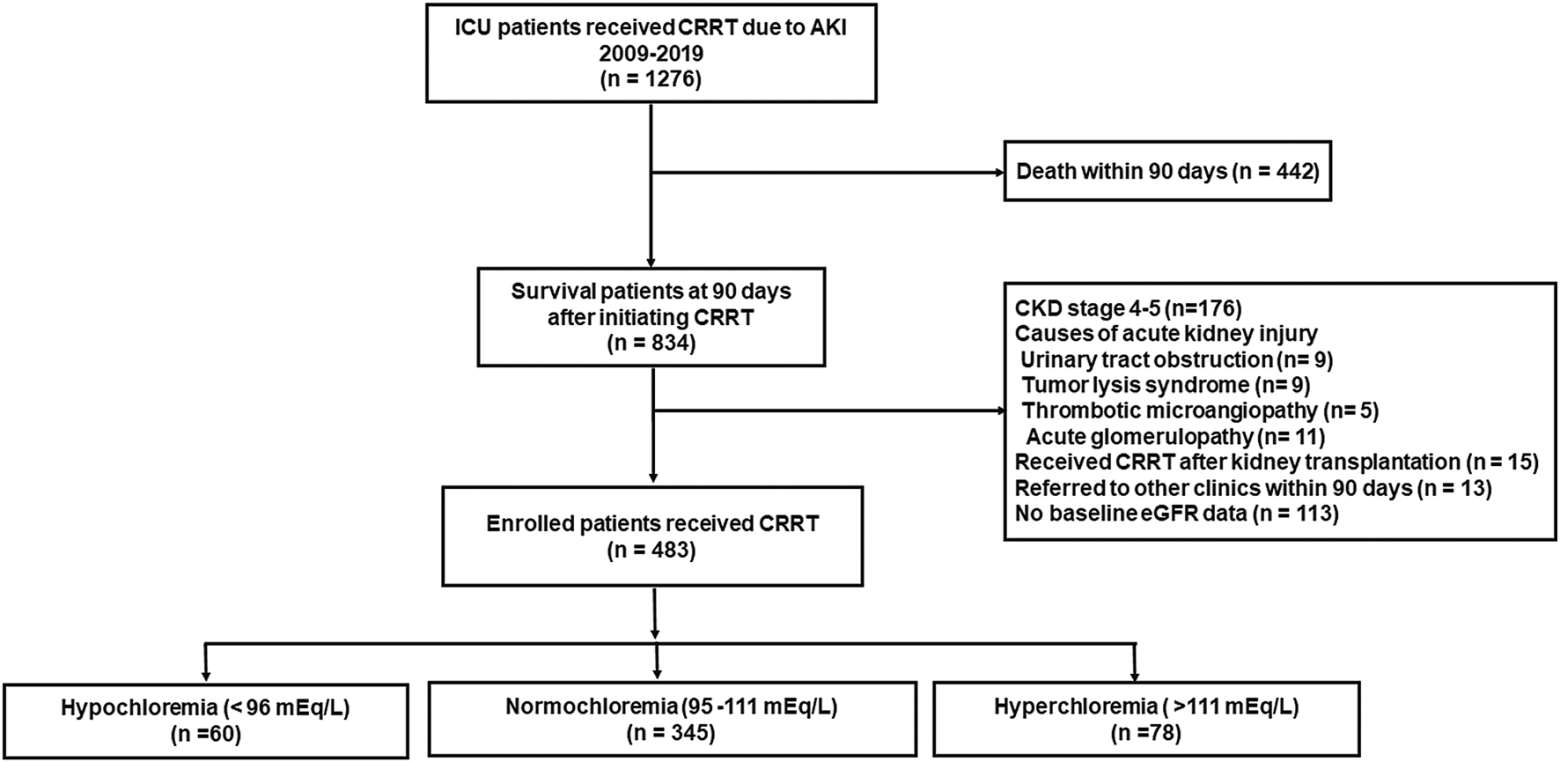
Patient inclusions and subgroupings.

During the 10-year study period, 1276 patients were included. Of 1276 patients, 834 patients survived at 90 days after initiating continuous renal replacement therapy (CRRT). Of 834 survivors, 351 were excluded: 176 patients with severe CKD, 34 patients whose cause of AKI was urinary tract obstruction, tumor lysis syndrome, thrombotic microangiopathy or acute glomerulopathy, 15 patients who received CRRT after kidney transplantation, 13 patients who were referred to other clinics within 90 days, and 113 patients without records in the laboratory database within 12 months before hospital admission (Fig. 1).

### Characteristics at the time of CRRT

Overall, 483 patients with AKI requiring CRRT were included in this study, of whom 293 were men. The mean age of the patients was 65.6 years, and sepsis in 154 patients (31.9%) was the most common cause of AKI at the time of CRRT initiation. The median Charlson Comorbidity Index (CCI), Sequential Organ Failure Assessment (SOFA) score, and mean simplified acute physiology score III (SAPS III) were 2.0 (0.0–3.0), 9 (8.0–11.0), and 43.9 ± 8.5, respectively. The patients were divided into three groups according to the degree of dyschloremia (hypochloremia, normochloremia, and hyperchloremia); 60 (12.4%) were in the hypochloremia group, 345 (71.4%) were in the normochloremia group, and 78 (16.1%) were in the hyperchloremia group. Although patients with hypochloremia had comparable CCIs to patients with normochloremia, those with hypochloremia had a higher prevalence of congestive heart failure than those with normochloremia. Meanwhile, patients with hypochloremia and hyperchloremia had higher SAPS III than patients with normochloremia (Table 1). Of note, all patients had blood sampling at 90 days after CRRT initiation.

**Table 1:** Patients' characteristics according to chloride category.

Variable	Hypochloremia (n = 60)	Normochloremia (n = 345)	Hyperchloremia (n = 78)	*P*-value
**Demographic characteristics**				
Age (years)	63.5 (57.0–72.0)	66.0 (55.0–75.0)	67.5 (55.0–79.0)	0.23
Male, n (%)	37 (61.7)	212 (61.4)	44 (56.4)	0.70
**Comorbid disease**				
CKD, n (%)	11 (18.3)	42 (12.2)	7 (9.0)	0.25
MI, n (%)	5 (8.3)	39 (11.3)	8 (10.3)	0.78
CHF, n (%)	20 (33.3)	48 (13.9)	8 (10.3)	< 0.001
CVA, n (%)	6 (10.0)	59 (17.1)	12 (15.4)	0.38
PVD, n (%)	3 (5.0)	16 (4.6)	4 (5.1)	0.98
Dementia, n (%)	6 (10)	21 (6.1)	5 (6.4)	0.53
DM, n (%)	7 (11.7)	27 (7.8)	7 (9.0)	0.61
Cirrhosis, n (%)	4 (6.7)	19 (5.5)	2 (2.6)	0.49
COPD, n (%)	2 (3.3)	10 (2.9)	4 (5.1)	0.61
Malignancy, n (%)	6 (10.0)	36 (10.4)	5 (6.4)	0.56
Charlson comorbidity index	2.0 (0.0–4.0)	2 (0–3.0)**	1.0 (0.0–2.0) ^#^	0.02
**Cause of AKI requiring RRT**				
Sepsis, n (%)	18 (30.0)	103 (29.9)	33 (42.3)	0.10
Ischemia, n (%)	14 (23.0)	93 (27.0)	16 (20.5)	0.46
Surgery, n (%)	3 (5.0)	47 (13.6)	5 (6.4)	0.06
Others, n (%)	25 (41.7)	102 (29.6)	24 (30.8)	0.17
**Clinical data**				
Time to start CRRT (days)	1.0 (0.8–1.3)	1.1 (0.8–1.5)	1.1 (0.7–1.7)	0.27
SOFA score	9.0 (8.1–11.0)	9.0 (8.0–11.0)	10.0 (8.0–12.0)	0.158
SAPS III	48.9 ± 6.4*	42.6 ± 8.5**	45.8 ± 8.3^#^	< 0.001
Urine output (mL/h)	7 (5–8)	6 (5–8)	6 (5–8)	0.229
Loop diuretic therapy (%)	55 (91.7)	309 (89.6)	63 (80.8)	0.06
Fluid balance (L/day)	1.8 (1.2- 2.0)	1.9 (1.5–2.1)	1.8 (1.4–2.2)	0.088
Chloride administration (mEq/day)	295.3 (215.0–340.3)	317.2 (263.0–373.0)	321.9 (232.8–370.5)	0.07
**Biochemical data**				
WBC (10^3^/mm^3^)	10.2 (6.9–16.1)	11.3 (7.7–16.6)	10.9 (8.0–17.9)	0.69
Hct (%)	28.0 (23.8–33.5)	28.4 (24.0–32.8)	29.1 (22.5–34.8)	0.80
Platelet (10^3^/mm^3^)	159.0 (91.0–233.0)	123.0 (68.0–200.0)	123.5 (77.0–187.0)	0.09
BUN (mg/dL)	52.5 (20.2–79.8)	49.1 (29.3–72.4)	46.3 (25.6–67.1)	0.75
Baseline Cr (mg/dL)	1.1 (0.8–1.3)	1.0 (0.8–1.3)	1.1 (0.9–1.3)	0.08
Baseline eGFR (mL/min per 1.73 m^2^)	65.2 (62.2–69.1)	67.2 (63.3–81.0)	66.5 (63.2–72.7)	0.26
Cr (mg/dL)	5.2 (3.2–8.5)*	4.2 (2.6–6.6)**	3.0 (2.1–4.7)^#^	< 0.001
eGFR (mL/min per 1.73 m^2^)	9.5 (5.6–15.8)*	12.9 (7.5–23.4)**	19.2 (11.2–28.0)^#^	< 0.001
Na (mEq/L)	133 (129–138)*	137 (134–141)**	144 (140–149)^#^	< 0.001
K (mEq/L)	4.5 (4.1–5.5)	4.4 (3.8–5.2)	4.4 (3.7–5.1)	0.38
TCO_2_ (mmol/L)	17.4 ± 6.5	17.7 ± 5.0**	14.8 ± 4.6^#^	< 0.001
Total bilirubin (mg/dL)	0.5 (0.4–1.1)	0.7 (0.5–1.3)	0.7 (0.5–1.2)	0.06

CKD, chronic kidney disease; MI, myocardial infarction; CHF, congestive heart failure; CVA, cerebrovascular accident; PVD, peripheral vascular disease; COPD, chronic obstructive pulmonary disease; AKI, acute kidney injury; CRRT, continuous renal replacement therapy; SOFA, Sequential Organ Failure Assessment; SAPS III, Simplified Acute Physiology Score III; CCI, Charlson comorbidity index; SBP, systolic blood pressure; DBP, diastolic blood pressure; MAP, mean arterial pressure; WBC, white blood cell; Hct, hematocrit; Cr, creatinine; BUN, blood urea nitrogen; eGFR, estimated glomerular filtration rate; Na, sodium; K, potassium; TCO_2_, total carbon dioxide. Categorical variables were compared by chi-square test or Fisher’s exact test. Continuous variables were compared by Analysis of variance (AVONVA) or the Kruskal–Wallis test. Of note, Bonferonni post hoc tests or Dunn procedure was used for pairwise comparisons.

*, *P* < 0.05 versus normochloremia group; **, *P* < 0.05 versus hyperchloremia group; #, *P* < 0.05 versus hypochloremia group. Values have been expressed as means ± standard deviations or as numbers (percentages).

### Kidney outcome according to chloride category

**Figure 2:**
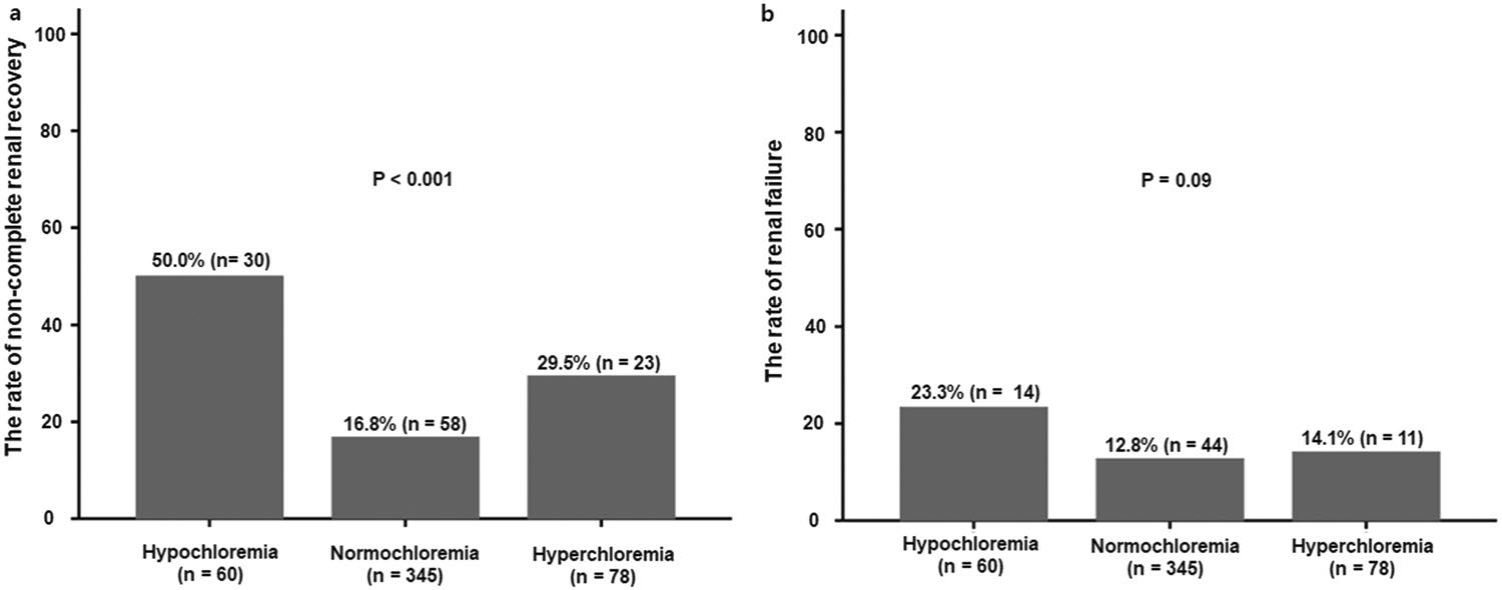
The rate of failure to restore renal function. (**a**) The incidence of non-complete renal recovery was significantly lower in the normochloremia group than in the other groups (*P* < 0.001). (**b**) There was no significant difference in the incidence of renal failure among three groups (*P* = 0.09). Categorical variables were compared by chi-square test or Fisher’s exact test.

Of 483 patients, 111 (23%) had non-complete renal recovery and 69 (14.3%) had renal failure at 90 days after initiating CRRT. The incidence of non-complete renal recovery was significantly lower in the normochloremia group than in the other groups (*P* < 0.001). Although there was no significant difference in the incidence of renal failure among three groups, a similar pattern was observed in the development of renal failure (Fig. 2).

### Factors associated with renal outcome

In logistic regression analysis, CKD, CHF, high CCIs, high creatinine concentrations, and dyschloremia were independently associated with non-complete renal recovery. Specifically, multivariate logistic regression analysis showed that hypochloremia and hyperchloremia were significantly associated with a > fivefold (odds ratio, 5.12; 95% confidence interval [CI], 2.56–10.23; *P* < 0.001) and a twofold (odds ratio, 2.53; 95% CI, 1.25–5.13; *P* = 0.01) higher risk of non-complete renal recovery, respectively (Table 2). Meanwhile, dyschloremia was also independently associated with the development of renal failure. However, only hypochloremia was significantly associated with a > twofold (odds ratio, 2.74; 95% CI, 1.19–6.32, *P* = 0.02) higher risk of renal failure (Table 3).

**Table 2:** Factors associated with non-complete renal recovery.

Variable	Univariate analysis		Multivariate analysis^a^	
	OR (95% CI)	*P*-value	OR (95% CI)	*P*-value
Age (per 1 year)	1.01 (0.99–1.02)	0.35	1.01 (0.99–1.03)	0.23
Male (vs. Female)	0.70 (0.46–1.08)	0.11	0.66 (0.41–1.08)	0.10
**Risk factors for deteriorating kidney function**				
CKD	2.80 (1.59–4.93)	< 0.001	2.12 (1.10–4.08)	0.02
CHF	2.12 (1.25–3.59)	0.01	1.36 (0.71–2.59)	0.36
CCI	1.18 (1.07–1.29)	0.001	1.15 (1.02–1.28)	0.02
**Cause of AKI required CRRT**				
Sepsis	1.21 (0.77–1.89)	0.40		
Surgery	0.93 (0.47–1.83)	0.83		
Ischemia	0.87 (0.53–1.43)	0.57		
Other	0.96 (0.61–1.52)	0.87		
**Clinical data**				
SOFA score	1.08 (0.90–1.19)	0.09	1.10 (0.99–1.22)	0.08
SAPS III	1.02 (0.99–1.05)	0.08	0.99 (0.95–1.03)	0.56
Urine output (per 1 mL/h)	1.05 (0.98–1.12)	0.16	1.06 (0.98–1.14)	0.15
Cr (per 1 mg/dL)	1.11 (1.04–1.18)	0.001	1.11 (1.02–1.20)	0.01
Na (per 1 mEq/L)	0.99 (0.98–1.02)	0.76	1.01 (0.97–1.04)	0.79
K (per 1 mEq/L)	0.98 (0.81–1.20)	0.87	0.78 (0.61–0.99)	0.04
TCO_2_ (per 1 mmol/L)	0.98 (0.94–1.02)	0.26	0.97 (0.93–1.02)	0.24
Total bilirubin (per 1 mg/dL)	0.96 (0.89–1.04)	0.35	0.99 (0.92–1.07)	0.84
**Dyschloremia**				
Normochloremia	Reference		Reference	
Hyperchloremia	2.07 (1.18–3.63)	0.01	2.53 (1.25–5.13)	0.01
Hypochloremia	4.95 (2.77–8.83)	< 0.001	5.12 (2.56–10.23)	< 0.001

CKD, chronic kidney disease; CHF, congestive heart failure; CCI, Charlson comorbidity index; SAPS III, Simplified Acute Physiology Score III; SOFA, Sequential Organ Failure Assessment; CRRT, continuous renal replacement therapy; AKI, acute kidney injury; Cr, creatinine; Na, sodium; K, potassium; TCO_2_, total carbon dioxide; OR, odds ratio; CI, confidence interval.

^a^Adjusted for age, male, CKD, CHF, CCI, SAPS III score, SOFA, urine output, Cr, Na, K, TCO_2_, total bilirubin, hyperchloremia, and hypochloremia.

**Table 3:** Factors associated with renal failure.

Variable	Univariate analysis		Multivariate analysis^a^	
	OR (95% CI)	*P*-value	OR (95% CI)	*P*-value
Age (per 1 year)	1.01 (0.99–1.02)	0.64	1.03 (1.00–1.05)	0.05
Male (vs. Female)	0.94 (0.56–1.58)	0.82	0.99 (0.55–1.76)	0.96
**Risk factors for deteriorating kidney function**				
CKD	2.54 (1.34–4.82)	0.004	1.60 (0.78–3.30)	0.20
CHF	2.38 (1.31–4.33)	0.04	1.83 (0.88–3.78)	0.11
CCI	1.20 (1.07–1.33)	0.001	1.18 (1.04–1.35)	0.01
**Cause of AKI required RRT**				
Sepsis	0.85 (0.49–1.49)	0.58		
Surgery	1.20 (0.56–2.58)	0.64		
Ischemia	0.95 (0.53–1.71)	0.87		
Other	1.12 (0.65–1.92)	0.69		
SAPS III	0.98 (0.96–1.02)	0.34	1.01 (0.95–1.04)	0.35
SOFA score	1.11 (0.99–1.24)	0.07	1.14 (1.00–1.31)	0.05
**Clinical data**				
Urine output (per 1 mL/h)	1.02 (0.94–1.10)	0.66	1.01 (0.92–1.10)	0.91
Cr (per 1 mg/dL)	1.15 (1.07–1.23)	< 0.001	1.11 (1.02–1.21)	0.02
Na (per 1 mEq/L)	0.99 (0.97–1.02)	0.81	1.02 (0.97–1.06)	0.48
K (per 1 mEq/L)	1.04 (0.83–1.32)	0.72	0.79 (0.59–1.03)	0.08
TCO_2_ (per 1 mmol/L)	0.95 (0.91–1.00)	0.06	0.93 (0.88–0.99)	0.01
Total bilirubin (per 1 mg/dL)	0.93 (0.82–1.05)	0.25	0.99 (0.89–1.09)	0.79
**Dyschloremia**				
Normochloremia	Reference		Reference	
Hyperchloremia	1.12 (0.55–2.29)	0.75	1.26 (0.53–3.01)	0.60
Hypochloremia	2.08 (1.05–4.10)	0.03	2.74 (1.19–6.32)	0.02

CKD, chronic kidney disease; CHF, congestive heart failure; CCI, Charlson comorbidity index; SAPS III, Simplified Acute Physiology Score III; SOFA, Sequential Organ Failure Assessment; CRRT, continuous renal replacement therapy; AKI, acute kidney injury; Cr, creatinine; Na, sodium; K, potassium; TCO_2_, total carbon dioxide; OR, odds ratio; CI, confidence interval.

^a^Adjusted for age, male, CKD, CHF, CCI, SAPS III, SOFA, urine output, Cr, Na, K, TCO_2_, total bilirubin, hyperchloremia, and hypochloremia.

### Association between serum chloride concentration as a continuous variable and renal outcome

**Figure 3:**
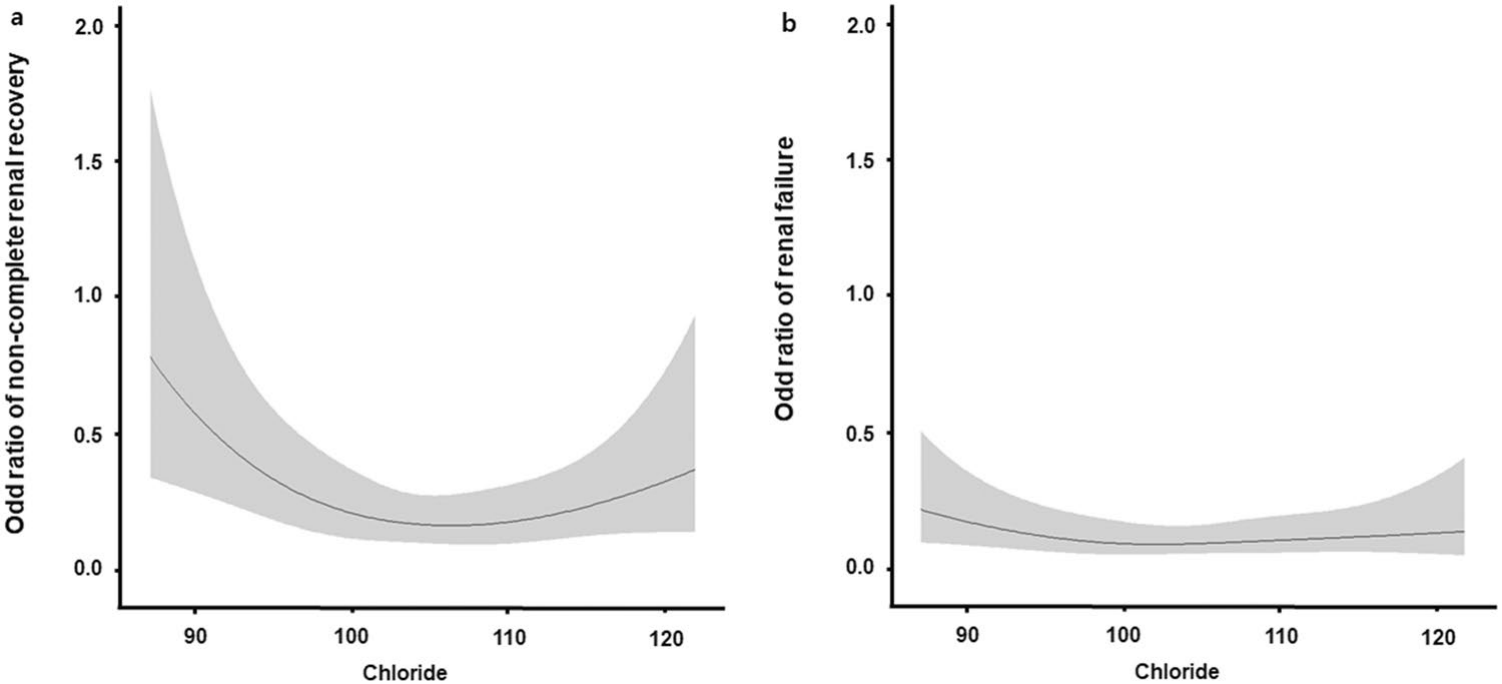
Restricted cubic spline plot of odds ratios for failure to restore renal function. Serum chloride concentrations have U-shaped associations with both (**a**) non-complete renal recovery and (**b**) renal failure. Multivariate logistic regression analysis adjusted for age, male, CKD, CHF, CCI, SAPS III, SOFA score, urine output, Cr, Na, K, TCO_2_, total bilirubin, hypochloremia, and hyperchloremia. CKD, chronic kidney disease; CHF, congestive heart failure; CCI, Charlson comorbidity index; SAPS III, Simplified Acute Physiology Score III; SOFA, Sequential Organ Failure Assessment, Cr, creatinine; Na, sodium; K, potassium; TCO_2_, total carbon dioxide.

Predicted odds ratios of renal outcome according to continuous values of serum chloride concentrations were generated using a cubic spline relationship. The graph shows a bidirectional or U-shaped association between serum chloride concentrations and non-complete renal recovery in the multivariate logistic regression model. In addition, when renal failure was considered as a renal outcome, a similar pattern was observed (Fig. 3).

## Discussion

This retrospective study showed that dyschloremia was independently associated with failure to restore renal function in survivors with severe AKI requiring RRT. Notably, when serum chloride concentrations were considered as continuous values, there was a U-shaped association between serum chloride concentrations and failure to restore renal function.

Severe AKI often requires renal replacement therapy in critically ill patients, which is known to be associated with a 20–50% mortality rate, development of CKD, and progression from CKD to renal failure^24^. Several epidemiologic studies showed that 10–30% of survivors with AKI requiring RRT remained dialysis-dependent^24,25^. In our study, the mortality rate (34.6%) and incidence of renal failure (13.7%) in patients with AKI requiring RRT were comparable to previous studies. In addition, electrolyte imbalances are very common clinical problems encountered in critically ill patients. Although the number of studies on chloride, known as forgotten anion, is limited, several studies have reported dyschloremia to also be very common (25–37%) in critically ill patients. Similar to previous studies, dyschloremia was also observed in 28.6% of the current study population (hypochloremia 12.5% and hyperchloremia 16.1%).

Hypochloremia in critically ill patients can be caused by diuretic therapy, significant gastric drainage, vomiting, chronic respiratory acidosis, heart failure, syndrome of inappropriate antidiuretic hormone secretion, and excess infusion of hypotonic solutions^26^. Moreover, excessive chloride administration during resuscitation with chloride-rich solutions, osmotic diuresis, fever, hypermetabolic states, and post hypocapnia are conditions causing hyperchloremia in critically ill patients^26^. Therefore, dyschloremia could be a sign of severe illness. Furthermore, in the current study, SAPS III was higher in patients with dyschloremia than in those with normochloremia.

After AKI occurs, the kidney can recover from cellular damage. However, renal function may not be fully restored, with the development of CKD or progression from CKD to renal failure^27^. Because proximal tubular epithelial cells in renal tissue are not only located closest to the glomeruli but also have the highest metabolic rate, they are vulnerable to injury^28^. If the regenerative processes of these injured cells remain incomplete, these cells interact with other cell types in the interstitium, leading to renal fibrosis and eventually CKD or renal failure^28^. Among patient-related factors in renal recovery following AKI, age, CKD, and comorbidity are the most frequently reported clinical factors^29^. Although illness severity scoring systems and organ dysfunction scores are thought to be additional clinical factors associated with renal recovery following AKI^29^, some studies have demonstrated that the prediction of renal recovery using these scoring systems was still ambiguous^30,31^. This study showed that high SAPS III and high SOFA scores were not associated with failure to restore renal function.

In this study, patients with dyschloremia had higher SAPS III than patients with normochloremia. However, dyschloremia was still independently associated with failure to restore renal function in survivors with severe AKI requiring RRT even after adjusting for other clinical factors such as SAPS III, suggesting that dyschloremia was associated with failure to restore renal function independent of the disease severity. Some studies found that hypochloremia was associated with increased intensive care units (ICU) length of stay or mortality^22,32^. Interestingly, in a study by Sho et al.^22^, compared with normochloremia, hypochloremia was an independent risk factor for the development of AKI. They hypothesized that the association of hypochloremia with AKI was mainly because of its association with potential hypovolemia, and not necessarily hypochloremia itself^22^. In contrast to the association of hypochloremia with renal function, the effects of hyperchloremia on renal function were well described. In vivo data suggested that vasoconstriction of the afferent arteriole of the renal glomerulus was induced by hyperchloremia^17^. In addition, an experimental study showed that renal blood flow and GFR were diminished after chloride infusion^33^. Furthermore, several clinical studies demonstrated that hyperchloremia was associated with the development of AKI^19,20^.

This study had some limitations. First, this was a small scale uncontrolled retrospective study. Therefore, there might be unmeasured confounding factors. Second, the study’s design cannot elucidate whether dyschloremia has a causal role or is merely a consequence of certain conditions. Third, although there have been several studies exploring the effects of dyschloremia on renal function, to the best of our knowledge, there have not yet been any experimental studies explaining the role of chloride in the recovery of renal function following AKI. Fourth, because CRRT usually corrects dyschloremia within a few days, the effect of dyschloremia on renal function is disputable in this study.

In conclusion, despite the current study’s limitations, it was the first to find an independent association of hypochloremia or hyperchloremia with failure to restore renal function following AKI. To clarify the role of dyschloremia in renal recovery following AKI, further well-designed clinical and experimental studies are needed.

## Methods

### Study design and patients

This observational retrospective study was conducted in 40-bed medical and surgical ICUs at university-affiliated hospitals from October 2009 to September 2019. Patients were eligible if they were aged 18 years or older, were admitted to the ICU, and received CRRT for severe AKI. In contrast, patients were excluded if they died or were referred to other clinics within 90 days after initiating CRRT, had pre-existing severe CKD (stage 4 or 5), had AKI caused by urinary tract obstruction, tumor lysis syndrome, thrombotic microangiopathy, or acute glomerulopathy, or received CRRT immediately after a kidney transplant. Of note, because treatments for tumor lysis syndrome, thrombotic microangiopathy, or acute glomerulopathy had direct effect on renal recovery, patients with these diseases were excluded. In addition, patients who did not have a record in the laboratory database within 12 months before hospital admission were also excluded.

### Definitions

First, hypochloremia, normochloremia, and hyperchloremia were defined as serum chloride concentrations of < 96 mEq/L, 96–111 mEq/L, and > 111 mEq/L, respectively. If at least one of the following criteria was met, i.e., a urine output < 0.3 mL/kg/h for ≥ 24 h, anuria for ≥ 12 h, increase in serum creatinine concentration ≥ threefold from baseline, or serum creatinine concentration ≥ 4 mg/dL with an acute rise of at least 0.5 mg/dL, a diagnosis of severe AKI was made. Second, baseline serum creatinine concentrations and estimated glomerular filtration rates (eGFR) were defined as average serum creatinine concentrations and eGFR within 12 months before hospital admission, respectively. Non-complete renal recovery was defined as a ≥ 25% decline in baseline eGFR. Finally, renal failure was defined as a persistent dialysis-dependent state.

### Continuous renal replacement therapy

The nephrologists in charge of the patients decided to initiate CRRT for patients with hyperkalemia (potassium level > 6.5 mEq/L), metabolic acidosis (pH < 7.15), or diuretic-refractory fluid overload (causing pulmonary edema). ICU nurses installed and maintained CRRT. In addition, the nephrologists considered stopping CRRT when the patients’ serum creatinine concentration had decreased and if there was a return to a spontaneous urine output of 1000 mL per 24 h. The femoral, internal jugular, or subclavian veins were utilized for CRRT. Although CRRT could not be discontinued, the patients’ dialysis mode was changed to intermittent hemodialysis if the patients were hemodynamically stable. All patients received continuous veno-venous hemodiafiltration using Prismaflex machines (Baxter, IL, USA) or Multifiltrate machines (Fresenius Medical Care, Bad Homburg, Germany). CRRT was initially started with a blood flow rate of 100 mL/min, which was gradually increased to 200 mL/min. The dialysate flow rate was 1000–2000 mL/h. The ultrafiltration dosage was set to at least 25 mL/kg/h.

### Data collection

The following data were collected from medical records at the time of CRRT; demographic data, causes of AKI requiring RRT, comorbidities, CCIs, clinical data such as SOFA scores and SAPS III, and biochemical data. Meanwhile, clinical data related to fluid balance, urine output, and chloride administration were collected between ICU admission and CRRT initiation. Cumulative fluid balance was defined as the difference between total intake and output during the period. Intake included fluids administered orally and parenterally, and output included urine, gastrointestinal losses, and drains. Total amount of chloride administration was calculated based on total intake. Cumulative fluid, total amount of chloride administrated, and total urine output were divided by the period. Furthermore, baseline serum creatinine concentrations and eGFR were collected as the average value for 12 months before hospital admission.

### Outcome evaluation

The study objective was to evaluate the incidence of dyschloremia at CRRT initiation and determine its association with renal recovery at 90 days after initiating CRRT. Renal recovery was classified into non-complete renal recovery and renal failure.

### Statistical analysis

Statistical analyses were performed using SPSS 19.0 (SPSS Inc., Chicago, Illinois, USA). Continuous variables are expressed as means ± standard deviations or medians and inter-quartile ranges according to the normality of distribution. Categorical variables are summarized as numbers (percentages). Continuous variables were compared using Analysis of variance (AVONVA) or the Kruskal–Wallis test. Of note, Bonferonni post hoc tests or Dunn procedure was used for pairwise comparisons. Categorical variables were compared using chi-square test or Fisher’s exact test. Logistic regression analysis was performed to assess variables associated with renal recovery. A multivariate analysis, which included all covariates with *P* values of < 0.1 in the univariate analysis, was performed. Although *P*-values were ≥ 0.1, potential confounding factors were included in the multivariate analysis. To better represent the shape of the association between serum chloride concentrations and renal recovery, serum chloride concentrations were considered to be continuous variables, and odds ratios were modeled using a restricted cubic spline curve.

### Ethical approval and informed consent

This study was performed in accordance with the Declaration of Helsinki and approved by the Institutional Review Board (IRB) of Kangdong Sacred Heart Hospital (Refs. Kangdong 2020-07-002). This was a retrospective medical record-based study, and the study subjects were de-identified. The IRB waived the need for written informed consent from the participants.

## Data Availability

The datasets generated during and/or analyzed during the current study are available from the corresponding author on reasonable request.
